# Synergistic impact of *Chlorella vulgaris*, zinc oxide- and/or selenium nanoparticles dietary supplementation on broiler’s growth performance, antioxidant and blood biochemistry

**DOI:** 10.1007/s11250-024-04098-5

**Published:** 2024-08-30

**Authors:** Rawda Sherif, Eldsokey Nassef, Seham El-Kassas, Abdulnasser Bakr, Elsayed Hegazi, Hanan El-Sawy

**Affiliations:** 1 Nutrition and Clinical Nutrition Department, Faculty of Veterinary Medicine, Kafrelsheikh University, Kafr El-Sheikh, Egypt; 2 Animal, Poultry, and Fish Breeding and Production, Department of Animal Wealth Development, Faculty of Veterinary Medicine, Kafrelsheikh University, Kafr El-Sheikh, 33516 Egypt

**Keywords:** Chlorella vulgaris, Zinc oxide, Selenium, Nanoparticles, Broilers, Performance

## Abstract

The current study explored the influence of dietary supplementation of *Chlorella vulgaris* dried powder (CV) with zinc-oxide-nanoparticles (ZnO-NPs), and/or selenium-nanoparticles (Se-NPs) on broilers’ growth, antioxidant capacity, immune status, histological responses, and gene expression of some related genes. Several 200 one-day-old Cobb-500 male chicks were distributed into 5 groups with four replicates each. In the 1st group, birds were fed the basal diet (BD). In the 2nd, 3rd, 4th, and 5th groups, birds received the BD supplemented with CV only, CV + ZnO-NPs, CV + Se-NPs, and CV + ZnO-NPs + Se-NPs, respectively. The CV dried powder, ZnO-NPs, and Se-NPs were added to the BD at a rate of 1 g, 40 mg, and 0.3 mg/kg diet, respectively. After 6 weeks of feeding, increases in final body weights (*P* < 0.05), body weight gain (*P* < 0.05), and feed intake (*P* < 0.05) were linked with improvements in FCR (*P* < 0.05) and intestinal morphometric indices (*P* < 0.05), and marked up-regulations of *MYOS* (*P* < 0.05), *GHR* (*P* < 0.05), and *IGF* (*P* < 0.05) genes were established. Additionally, distinct increases in antioxidant enzyme activities of SOD (*P* < 0.05), and GPX (*P* < 0.05) with increases in the mRNA copies of their genes were measured. Moreover, slight improvement in immunity indices, WBCs count (*P* > 0.05), and phagocytic and lysozyme activities (*P* > 0.05) were found. However, distinct increases in phagocytic index (*P* < 0.05) and up-regulations of *IL-1β* and *TNF*, and down-regulation of IL-10 mRNA levels were reported (*P* < 0.05). These findings were prominent in the case of the separate supplementation of CV with ZnO-NPs or Se-NPs confirming the synergistic mechanisms of CV with ZnO-NPs or Se-NPs. Thus, the synergetic supplementation of CV with ZnO-NPs, or Se-NPs in the broiler’s diet could augment their growth and antioxidant response.

## Introduction

Increasing poultry production is crucial to face the continuous increase in the human population especially in the case of global crises such as the Covid-19 pandemic, and the Russian-Ukraine war which increase the cost of traditional feed ingredients (Fathy Megahed et al. 2023). Therefore, using alternative feedstuff, and feed additives to enhance the efficiency of feed utilization and to reduce feed cost is crucial.

Microalgae such as *Chlorella vulgaris* (CV) have garnered attention as good alternative feed sources that are employed to yield varied series of essential nutrients such as carbohydrates, protein, fat, vitamins, and other organic minerals (Abdelnour et al. 2019; Amidi et al. 2016). It has been effectively used as a feed additive to positively improve the growth performance, immune response, and meat quality of poultry (Spínola et al. 2023). The reported improvement in birds’ performance is attributed to its bioactive components which exert antimicrobial and immunomodulatory activities (Tounsi et al. 2022). Besides, the CV’s high contents of chlorophyll, β-carotene, phenolic acid, γ-linolenic acid, flavonoid, phycobiliproteins, and vitamins C, and E are responsible for its antioxidant activity. The CV-associated antioxidant capacity neutralizes stressful molecules such as free radicals, preventing cellular damage due to oxidative stress in the case of chronic diseases and aging-related processes (Abdelnour et al. 2019). In addition, CV induces immunomodulatory impacts by regulating the immune response via enhancing the immune cells’ production and activities which help the body against pathogens and foreign substances (Riccio and Lauritano 2019).

Trace minerals such as zinc (Zn) and selenium (Se) are one of the feed ingredients that are indispensable to maintain the physiological metabolism of birds (Cruz and Fernandez 2011). They catalyze a wide variety of enzyme systems involved in carbohydrate metabolism (Alagawany et al. 2021; Brand and Kleineke 1996) and can help in regulating birds’ antioxidant response and immunity. However, because of the low bioavailability of the inorganic salts of these minerals and the emergence of nanotechnology, so, using nanoparticles of zinc and selenium can effectively improve their functions. This is because the nanotechnology allows reducing the size of these mineral particles into small nano size that increases its absorption, and bioavailability (El-Kassas et al. 2019). However, the climbing price of the nano-application might hinder its applications in the livestock industry, but its higher bioavailability and massive socio-economic and environmental benefits have been anticipated for nanotechnology and make them more effective (Ahmad et al. 2022; National Nanotechnology Initiative 2011).

Thus, in this nutrition experiment, we hypnotized that the combination of CV with ZnO-NPs and Se-NPs would maximize the benefits of CV supplementation and at the same time, get more benefits from ZnO-NPs and Se-NPs as well. Therefore, the current study aimed to explore the effect of the separate and synchronized supplementation of CV, Se-NPs, and/or ZnO-NPs in a broiler’s diet on its growth performance, immunological comeback, histological response, and gene expression of some related genes. For a comprehensive illustration of how the dietary supplementation of CV, and nanoparticles of zinc and selenium could impact broiler’s growth performance and antioxidant capacity, a deeper investigation into their influence on the expression levels of specifically related genes was performed. This represents a nutrigenomics approach to describe how the dietary supplementation of CV, and nanoparticles of zinc and selenium influences a bird’s genetic expression.

## Materials and methods

### Birds, housing, and diets

Two hundred one-day-old Cobb-500 male chicks were obtained from a commercial hatchery (Kafrelsheikh, Egypt) to be used in this study. To get rid of the transportation-associated stress, birds were reared together (for 2 days) in an environmentally controlled room (3 × 15 × 3 m^3^) at the poultry research facility at the Faculty of Veterinary Medicine, Kafrelsheikh University, and fed the basal diet (BD) without any supplementations (El-Naggar et al. 2019). At three days old, birds were individually weighed (the average initial body weight was 59.82 ± 1.32) and then randomly clustered into five treatment groups with four replicates each (*n* = 10 birds/replicate). Birds were housed in stocking floor pens of 1 × 1.5 m^2^ in size. The first group of birds was fed on the BD without any supplements, while birds in the 2nd, 3rd, 4th, and 5th groups were fed the BD supplemented with *Chlorella vulgaris* (CV) dried powder, CV and zinc oxide nanoparticles mixture (CV + ZnO-NPs), CV and Selenium-nanoparticles mixture (CV + Se-NPs), and the combination of the three supplements (CV + ZnO-NPs + Se-NPs), respectively. The CV dried powder, ZnO-NPs, and Se-NPs were added to the BD at rates of 1 g, 40 mg, and 0.3 mg/kg diet, respectively. The CV dried powder was obtained from the Algae Production Unit, National Research Institute, Dokki, Giza, Egypt. The chemical analysis of this CV to determine the amino acids content (kg DM) was previously conducted by (Kholif et al. 2017) and reported the presence of 105 g, 52·4 g, 50·8 g, 107 g, 51·0 g, 84·4 g, 64·4 g, 15·0 g, 50·1 g, 68·4 g, 52·0 g, 42·0 g, 50·2 g, 56·0 g, 82·0 g and 64·0 g of aspartic acid, threonine, serine, glutamic acid, glycine, alanine, valine, methionine, isoleucine, leucine, tyrosine, phenylalanine, histidine, lysine, arginine and proline, respectively. Besides, they reported the presence of 632·4 g and 367·3 g of total saturated fatty (SFA) and unsaturated fatty (UFA) acids, respectively. Moreover, the evaluation of phenolic contents of CV was performed by (Abu-Serie et al. 2018) and showed that the one gram of CV contains 0.36 ± 0.02, 0.16 ± 0.01, 5.18 ± 0.58, 3.46 ± 0.21, 1.02 ± 0.01, 5.45 ± 0.25, and 1.04 ± 0.05 (mg/g) of 2,5 dihydroxybenzoic acid, gallic acid, total phenolics, flavonoids, tannins, triterpenoids, and sulfated polysaccharides, respectively. Also, the mineral analysis revealed the presence of 30.08 ± 0.01, 279 ± 0.00, 4300 ± 0.01, 4180 ± 0.00, 20,000 ± 0.00, and 4260 ± 0.01 (µg/g) of Cu, Se, Zn, Fe, Ca, and Mg, respectively. All these constituents account for the antioxidant, anti-inflammatory, and antibacterial features of CV (Abu-Serie et al. 2018). The ZnO-NPs and Se-NPs were synthesized at the Nano Chemistry Lab., Chemistry Department, Faculty of Science, Kafrelsheikh University, Egypt using the precipitation method (El-Shafai et al. 2021).

The BD was formulated to meet the nutrient requirements of Cobb-500 according to the Cobb-500 nutrient requirements guide and NRC poultry requirements (NRC 1994). The ingredients and the chemical analysis of the diets (starter, grower, and finisher) (AOAC 2000) are presented in Table 1. The starter diet was offered to the birds in the first two weeks, and then the grower diet was offered in the next two weeks (3rd and 4th week) after that the finisher diet was offered to birds till the end of the experiment in the 6th week.

**Table 1:** Ingredients and calculated analysis of the diets

Ingredient (g/kg diet)	Starter	Grower	Finisher
Yellow corn	548.50	608.30	640.80
Soybean meal (44%)	332.70	280.00	247.00
Corn gluten meal	60.00	52.5	49.5
Vegetable oil	16.00	16.00	20.00
Limestone^1^	14.00	14.00	14.00
DCP ^2^	18.00	18.00	18.00
Lysine	3.00	3.40	2.90
Methionine	1.3	1.3	1.3
Choline chloride	0.5	0.5	0.5
Common salt	3	3	3
Mineral and Vitamin Premix^3^	3	3	3
*Chemical composition* (%)			
Crude protein %	22.24	20.00	18.00
Crude fiber %	3.37	3.09	3.00
Ether extract %	6.01	4.8	4.74
Metabolizable Energy (ME (Kcal/kg diet))	3000	3057	3102.5
Calorie/ protein ratio *	134.89	152.85	172.36
Calcium *	0.97	0.96	0.95
Available phosphorus*	0.49	0.46	0.43
Lysine *	1.37	1.26	1.15
Methionine *	0.51	0.50	0.46

^1^Limestone contains 35% calcium & locally produced. ^2^Di-calcium phosphate: contains 21% calcium and 17% phosphorus. ^3^Vitamin and mineral mix each 3kg contains: Vit A (12000000Iu), Vit D (2000000Iu), Vit E(10gm), vit K_3_ (2gm), vit B_1_ (1gm), vit B_2_ (5gm), vitB_6_ (1.5gm), vit B_12_ (10gm), nicotinic acid (30gm), pantothenic acid (10gm), folic acid (1gm), biotin (50 mg), choline chloride 50% (250gm), iron (30gm), copper (10gm), zinc (50gm), manganese (60gm), iodine (1gm), selenium (0.1gm), cobalt (0.1gm) and carrier up to 3kg. ^*^ calculated composition according to NRC (NRC 1994)

Bird’s management was performed to follow the recommended management by the Cobb-500 Broiler Management Guide, Cobb-Vantress, Inc., 2021. Accordingly, from the first day, the birds were brooded at 34 –32 °C which was gradually reduced to reach 22–25 °C at 21 days of age and continued to the end of the experiment. Additionally, the relative humidity was adjusted between 50 and 70% for all groups. The birds were housed at a stocking density of ten birds/m^2^ in floor pens of 1 × 1.5 m^2^ in size. Digital thermometers were used to check the house temperature and relative humidity. Birds received a 24-hour continuous light for the first week then subjected to 20:4 light and darkness, respectively until the end of the experiment. Feed and water were offered ad libitum. Besides, on the 7th, 18th, and 28th days, the birds were vaccinated with the Newcastle disease vaccination while on the 12th day, the infectious bursal disease vaccination was applied.

### Assessment of the growth performance

The birds’ body weights (BW), feed intake (FI), and body weight gains (BWG) were recorded biweekly. The feed conversion ratio (FCR) was then calculated from the feed intake data and calculated body gain based on the following formula calculated $\:\left(FCR\right)=\frac{Feed\:consumed\:\left(g\right)}{weight\:gain\left(g\right)}\:\:$(El-Katcha et al. 2023).

### Samples collection

At the end of the 6th week of the feeding trial, all birds were individually weighed to record the final live BW. Then 3 birds from each replicate (12 per treatment) were randomly selected for sample collection (blood and tissues). Two blood samples were collected from each bird from the jugular vein in heparinized and non-heparinized syringes for whole blood and serum, respectively. The serum was separated by the centrifugation of the blood samples at 3000 rpm for 10 min then the serum was stored at − 20 ºC for the biochemical analysis. Birds were then killed by cervical dislocation under mild anesthesia. Specimens from the liver, spleen, and muscle were collected, for histological and gene expression analysis. The tissue specimens for the gene expression analysis were quickly frozen in liquid nitrogen and then stored at − 80ºC until used for RNA extraction.

### Whole blood and serum biochemical parameters

The collected whole blood samples were used to evaluate the red blood cell counts (RBCs) (10^6^ /mm^3^), hemoglobulin content (HB) (g/100mL), packed cell volume (PCV) (%), MCHC, MCH, and MCV. Besides, the differential WBCs count, basophils (10^6^ /mm3), monocytes (10^6^ /mm3), lymphocytes (10^6^ /mm3), and heterophils (10^6^ /mm3) were assessed for all groups. Then heterophils/ lymphocytes (H/L) ratio was determined. The WBCs and RBCs counts were done manually using a hemocytometer based on the method described by (Campbell 1995). Liver function indicators; alanine aminotransferase (ALT), and aspartate Aminotransferase (AST) besides, the kidney function indices; urea, and creatinine were measured in the serum samples for all groups. In addition, the total protein, albumen, and globulin were also, evaluated. All the parameters were assessed by a spectrophotometric method using standard commercial kits according to the recommended ins3.tructions (Biodiagnostic Co, Egypt).

### Determination of antioxidant enzyme activities, phagocytic functional assay, and serum lysozyme activity

The activities of antioxidant enzymes such as superoxide dismutase (SOD), catalase (CAT), and glutathione peroxidase (GPx) were assessed in the serum samples by a UV-VIS spectrophotometer using commercial kits (Biodiagnostic, Egypt) following the recommended manufacturing procedures. The phagocytic functional assay to determine the phagocytic activity and phagocytic index was done in vitro, using *Candida albicans* following the methods formerly done by (El-Kassas et al. 2018). Briefly, equivalent amounts of fresh whole blood, *C. albicans* suspension (containing 1 × 10^6^ cells), and fetal bovine serum (South American) were mixed and then incubated at 37 °C for 30 min. The cells were then harvested by centrifugation at 1500 rpm for 10 min and then resuspended. After that blood smears were prepared by 5µL of cells and stained by a commercial rapid field stain containing polychrome methylene blue and eosin. The blood smears were washed and dried before the microscopical examination. The phagocytic activity (PA) was estimated as a percentage of phagocytic cells that engulfed yeast cells while the phagocytic index (PI) equals the total number of yeast cells phagocytized divided by the number of phagocytic cells (El-Kassas et al. 2018).

Lysozyme activity was also, assessed in all collected serum samples based on the method described by (El-Kassas et al. 2018). In this method, the agarose gel (1%) was dissolved in a phosphate buffer (50 mM, pH 6.3) and then mixed with a suspension of *Micrococcus lysodeicticus* culture (500 mg / L). The mixture was then dispensed into Petri’s dish (14 cm diameter) and left to solidify at room temperature. Afterward, equal volumes (25 µl) of serum and a standard (hen egg white lysozyme solution, 20 mg / mL) were put into depressions in the agar and then incubated for 18 h at 37 °C. The diameters of the lysed zones were then measured (millimeters). The lysozyme activity was calculated by the logarithmic regression analysis, according to the following equation Y = A + B log X, where Y donates the diameter of the lysed zone while the X represents the lysozyme activity (µg/mL). The *C. albicans* and *Micrococcus lysodeicticus* mixtures were obtained from the Animal Health Research Institute, El-Dokki, Giza, EGYPT.

### Histomorphometry determination

For the histomorphological evaluation, specimens from the jejunum, spleen, and liver were sampled from the different experimental groups. Specimen preparations followed the previously described method by (Gewaily and Abumandour 2021). Briefly, these tissue specimens were first fixed in 10% formalin for 24 h and then transferred to alcohol (70%). After fixation, the tissue specimens were dried in ascending series of graded ethanol, which were then cleared in xylene. After that, the fixed specimens were infused and embedded in paraffin wax. Sections of 5 μm were cut by a Leica rotatory microtome (RM 20,352,035; Leica Microsystems, Wetzlar, Germany) and then were mounted on glass slides. Deparaffinization in xylene was done for the prepared tissue sections which then were rehydrated in descending graded series of ethanol and at the end in distilled water. Finally, the slides were stained using conventional staining using hematoxylin and eosin (H&E). The stained slide sections were examined using a light microscope (Leica DM500; Leica Microsystems, Japan). For the jejunum, the length, and width of the jejunal villi as well as its crypt depth were evaluated using the Image J software (Image J software, Bethesda, MD, USA) as described in (Kirrella et al. 2023a). In this regard, five fields for each slide and ten intact villi and the space between two consecutive villi in each field were included in the assessment of the jejunal villi length, and width as well as its crypt depth.

### Real-time PCR

To assess the gene expression levels of some related genes in response to the dietary supplementation of CV, CV + ZnO-NPs CV + Se-NPs, and CV + ZnO-NPs + Se-NPs mixture, total RNA was extracted from 30 to 50 mg of liver, spleen, and muscle tissue samples using TRIzol (Applied Biosciences) according to the manufacturer’s guidelines. The RNA was then evaluated for its integrity using ethidium bromide-stained gel electrophoresis (2% agarose gel) through the visual evaluation of rRNA bands (18 S and 28 S). After that, the Nanodrop (UV-Vis spectrophotometer Q5000, Quawell, USA) was used to evaluate the concentration of the RNA. Afterward, a fixed volume (about 2 µg) of RNA was used in reverse transcription using a cDNA synthesis commercial kit (SensiFAST™ cDNA Synthesis Kit (Bioline, United Kingdom). Briefly, a fixed volume of RNA (2 µg) was mixed with 4 µl of transAmp buffer, 1 µl Reverse Transcriptase, and DNase/RNase free water was completed up to 20 µl. The mixture was gently mixed by pipetting and incubated at 25 ^o^C for 10 min (primer annealing), 42 ^o^C for 15 min (reverse transcription), 85 ^o^C for 5 min (inactivation), then held at 4 C. Then, the produced cDNA was used to assess the gene expression profile of the studied genes using specific primers (Table 2). The qPCR reactions were done using the SensiFast™ SYBR Lo-Rox master mix (Bioline, United Kingdom) in a MxPro-qPCR system (Agilent Technologies, USA). The qPCR mixture was prepared by adding 10 µl of SensiFast™ SYBR master mix, and 0.5 µM from each primer to 2 µl of cDNA samples. The reaction was completed under the following thermal cycling conditions: initial denaturation for 15 min at 95^o^C, then, 40 cycles included denaturation at 95^o^C for 15 s, annealing for 1 min at gene-specific temperatures. The specificity of the PCR products was confirmed by analyzing the dissociation curves (melting curves) starting at 65^o^C to 95^o^C, with an increase of 0.5^o^C every 5 s. The specific amplified PCR product displayed only one peak at a specific melting temperature. All studied genes were assessed in duplicates for all samples (*n* = 12/treatment). The relative mRNA levels (fold change) of the assessed genes for each sample were calculated using the obtained CT values according to the Livak method (Livak and Schmittgen 2001). In this regard, the relative mRNA expression levels for each gene in every sample were normalized against the housekeeping gene (β actin and GAPDH) and the CT values of the control group (fed BD only). In brief, the ΔCTs of each sample were calculated by subtracting the CT values of the housekeeping gene from that of the tested gene (CT_tested gene_ – CT _housekeeping gene_). Then the ΔΔCT was calculated by subtracting the average ΔCT of the control samples from the ΔCT of each sample (ΔCT _treated samples_ – ΔCT _control_). After that, the relative mRNA levels were expressed as fold change calculated from 2^− ΔΔCT^.

**Table 2:** Primer sequences (5’-3’) used in real-time PCR

Gene	Primer	Annealing (^o^C)	Accession number	Amplicon size	References
*β -actin*	F: ACCTGAGCGCAAGTACTCTGTCT R: CATCGTACTCCTGCTTGCTGAT	60	NM_205518.1	95	(Xie et al. 2014)
*GAPDH* ^*1*^	F: GGGCACGCCATCACTATCTTC R: ACCTGCATCTGCCCATTTGAT	60	NM_204305	86	(Nerren et al. 2009)
*GHR* ^*2*^	F: AACACAGATACCCAACAGCC R: AGAAGTCAGTGTTTGTCAGGG	60	KF957983.1	145	(El-Naggar et al. 2019)
*IGF* ^*3*^	F: CACCTAAATCTGCACGCT R: CTTGTGGATGGCATGATCT	60	NM_001004384.2	140	(El-Naggar et al. 2019)
*MYOS* ^*4*^	F: GCAAAAGCTAGCAGTCTATG R: TCCGTCTTTTTCAGCGTTCT	63	NM_001001461.2	109	(Sakr et al. 2020)
*SOD* ^*5*^	F: CGGGCCAGTAAAGGTTACTGGAA R: TGTTGTCTCCAAATTCATGCACATG	60	NM_205064.2	83	(Abdo et al. 2017)
*GPx* ^*6*^	F: GCGACTTCCTGCAGCTCAACGA R: CGTTCTCCTGGTGCCCGAAT	60	NM_001277853.3	99	(El-Kassas et al. 2019)
*IL-1B* ^*7*^	F: CAGCCTCAGCGAAGAGACCTT R: CACTGTGGTGTGCTCAGAATCC	60	NM_204524.2	85	(El-Kassas et al. 2018)
*TNF-α* ^*8*^	F: CGCTCAGAACGACGTCAA R: GTCGTCCACACCAACGAG	60	MF000729.1	115	(Hao et al. 2021)
*IL10* ^*9*^	F: CAGACCAGCACCAGTCATCA R: TCCCGTTCTCATCCATCTTCTC	62	NM_001004414.4	163	(Adu-Asiamah et al. 2021)

^1^*GAPDH* = glyceraldehyde 3-phosphate dehydrogenase; ^2^*GHR*= growth hormone receptor ; ^3^*IGF*= Insulin like growth factor ; ^4^*MYOS* = myostatin gene; ^5^SOD=Superoxide Dismutase; ^6^ GPx1 = Glutathione peroxidase; ^*7*^*IL-1B = interleukins 1 beta.*^*8*^*TNF-α = Tumor necrosis factor alpha.*^*9*^*IL10 = interleukins 10*

### Statistical analysis

The obtained results were statistically analyzed using a one-way ANOVA in the SPSS package (©IBM Corp. Released 2013, IBM SPSS Statistics for Windows, Version 22.0. Armonk, NY: IBM). Then, Tukey’s multiple comparison tests were performed for multiple comparisons. The results were considered statistically significant at *P* < 0.05. The results were presented as the mean ± standard error of the mean (SEM). GraphPad Prism 9 (©GraphPrism Software, La Jolla, CA, USA) was used to create the figures.

## Results

### Growth performance

The feed intake and growth indices of Cobb-500 fed on BD supplemented with *Chlorella vulgaris* (CV) only, CV with ZnO-NPs, CV with Se-NPs, or their combination are described in Table 3. Combining the CV with ZnO-NPs or Se-NPs significantly increased Cobb’s final body weights to 2796 ± 69.19 g, and 2850 ± 62.50 g, respectively compared with 2497 ± 66.38 g, 2568 ± 64.13 g, and 2614 ± 52.33 g for control, CV, and CV + ZnO-NPs + Se-NPs. Also, the separate combination of BD with ZnO-NPs or Se-NPs significantly increased the total body gain to 2740 ± 82.56 g, and 2791 ± 67.76 g, respectively compared to 2438 ± 70.27 g for birds fed basal diet only (control), 2511 ± 79.05 g for those fed BD supplemented with CV only (CV), and 2555 ± 57.79 g in the case of the composite of the three substances (CV + ZnO-NPs + Se-NPs) (*P* < 0.05). However, the earlier body weights and gain (from 0 to 2 weeks, 2–4 weeks, 4–6 weeks) did not show any significant changes (*P* > 0.05).

**Table 3:** Growth performance of *chicken* after 6 weeks of separate and concurrent supplementation of CV, ZnO-NPs, and/or Se-NPs

	Control	CV	CV + ZnO-NPs	CV + Se-NPs	CV + ZnO-NPs + Se-NPs	*P* values
**Body weight (g)**						
Initial wt (0 wk)	60.83 ± 0.75	59.79 ± 1.27	58.68 ± 1.44	59.74 ± 1.65	60.08 ± 1.47	0.859
2 wk	453.64 ± 8.00	450.00 ± 7.07	445.83 ± 10.55	460.91 ± 11.14	440.83 ± 9.81	0.649
4 wk	1677.27 ± 35.76	1660.91 ± 62.01	1718.33 ± 38.12	1822.73 ± 42.92	1677.27 ± 41.76	0.114
6 wk	2497 ± 66.38 ^**b**^	2568 ± 64.13 ^**b**^	2796 ± 69.19 ^**a**^	2850 ± 62.50 ^**a**^	2614 ± 52.33 ^**b**^	0.0006
**Body weight gain (g)**						
0–2 wk	393 ± 7.78	388.74 ± 7.29	390.08 ± 11.48	401.55 ± 10.82	381.27 ± 10.36	0.721
2–4 wk	1223.64 ± 39.30	1202.00 ± 36.62	1293.64 ± 29.67	1361.82 ± 49.19	1236.36 ± 47.53	0.164
4–6 wk	876.00 ± 85.54	927.78 ± 81.44	1056.36 ± 76.19	1027.27 ± 67.62	990.00 ± 69.82	0.642
0-6wk	2438 ± 70.27 ^**b**^	2511 ± 79.05 ^**b**^	2740 ± 82.56 ^**a**^	2791 ± 67.76 ^**a**^	2555 ± 57.79 ^**b**^	0.0034
**Feed intake (g)**						
0–2 wk	589.06 ± 44.88	603.90 ± 53.76	778.46 ± 68.92	586.80 ± 52.45	579.35 ± 49.47	0.124
2–4 wk	1673.27 ± 94.41	1620.67 ± 87.96	1743.81 ± 99.85	1908.00 ± 93.07	1589.51 ± 95.05	0.065
4–6 wk	1736.67 ± 56.95 ^**b**^	1954.43 ± 98.52 ^**ab**^	2014.7 ± 97.66 ^**a**^	1974.20 ± 93.73 ^**a**^	1864.14 ± 91.44 ^**a**^	0.045
0-6wk	3999 ± 104.2 ^**b**^	4179 ± 154.8 ^**ab**^	4537 ± 172.9 ^**a**^	4469 ± 114.8 ^**a**^	4033 ± 27.09 ^**b**^	0.0367
**Feed conversion ratio (FCR) (g feed/ g gain)**						
0–2 wk	1.51 ± 0.03	1.56 ± 0.03	2.02 ± 0.07	1.47 ± 0.05	1.53 ± 0.05	0.962
2–4 wk	1.38 ± 0.05	1.41 ± 0.12	1.36 ± 0.03	1.42 ± 0.06	1.31 ± 0.05	0.739
4–6 wk	2.65 ± 0.54	2.32 ± 0.25	2.03 ± 0.17	2.02 ± 0.15	1.97 ± 0.14	0.427
0–6 wk	1.83 ± 0.066 ^**a**^	1.61 ± 0.022 ^**b**^	1.70 ± 0.053 ^**ab**^	1.61 ± 0.036 ^**b**^	1.65 ± 0.033 ^**b**^	0.046

These changes in body weight and weight gain were associated with modulations of FI and FCR. Marked increases in the total FI (0–6 weeks) were noticed in the case of CV, CV + ZnO-NPs, and CV + Se-NPs (4179 ± 154.8 g, 4537 ± 172.9 g, and 4469 ± 114.8 g, respectively) compared to BD only (3999 ± 104.2 g) and the concurrent combination of the CV, Nano-Zn, and Nano-Se (40.33 ± 27.09 g) (*P* < 0.05). Accordingly, the FCR was altered; all the supplemented groups exhibited significantly better FCR compared to the non-supplemented group (BD only) (*P* < 0.05).

### Blood biochemistry and hematology profiles

Feeding of diet supplemented with CV, CV + ZnO-NPs, CV + Se-NPs, or their combination did not alter the levels of total protein, globulin, and albumin as well as kidney function indicators (urea and creatinine) (*P* > 0.05) (Table 4). On the other hand, all the studied dietary supplements significantly lowered the levels of liver function indicators (ALT and AST) from 53.13 ± 2.14 & 34.018 ± 1.983, respectively in the non-supplemented control group to 39.035 ± 0.559 & 19.63 ± 1.009 in the case of the combination of BD with ZnO-NPs and Se-NPs (*P* < 0.05). Besides, the dietary supplementations of CV, CV + ZnO-NPs, CV + Se-NPs, and their combination were linked with significant increases in RBCs count and HB contents (Table 5) compared with the non-supplemented group (*P* < 0.05). For RBCs increased from 2.50 ± 0.198 in the control to 3.06 ± 0.365, 2.66 ± 0.181, 2.65 ± 0.227, and 3.75 ± 0.385 in supplemented groups, respectively, and for HB increased from 8.588 ± 0.253 in the control to 9.900 ± 0.712, 9.903 ± 0.462, 9.617 ± 0.612, 11.153 ± 0.675 for supplemented groups, respectively. No changes in the levels of MCHC, MCH, MCV, and WBCs were found in the supplemented groups compared to the control one (*P* > 0.05).

**Table 4:** Blood biochemistry indices and lipid profile

	Control	CV	CV + ZnO-NPs	Ch + Se-NPs	CV + ZnO-NPs + Se-NPs	*P* values
Total protein (g/dL)	2.77 ± 0.082	3.22 ± 0.252	3.28 ± 0.436	3.067 ± 0.068	3.55 ± 0.186	0.202
Globulin (g/dL)	1.35 ± 0.098	1.62 ± 0.230	1.72 ± 0.382	1.51 ± 0.052	1.91 ± 0.166	0.400
Albumin (g/dL)	1.66 ± 0.021	1.60 ± 0.022	1.56 ± 0.057	1.56 ± 0.034	1.65 ± 0.021	0.084
AST (U/L) ^1^	53.13 ± 2.14 ^**a**^	41.10 ± 0.636 ^**bc**^	48.001 ± 1.66 ^**b**^	44.96 ± 2.08 ^**b**^	39.035 ± 0.559 ^**c**^	< 0.0001
ALT (U/L) ^2^	34.018 ± 1.983 ^**a**^	25.408 ± 2.924 ^**b**^	19.673 ± 0.442 ^**b**^	20.93 ± 0.402 ^**b**^	19.63 ± 1.009 ^**b**^	< 0.0001
Urea (mg/dL)	3.955 ± 0.143	4.075 ± 0.286	3.821 ± 0.202	3.578 ± 0.203	3.775 ± 0.251	0.622
Creatinine (mg/dL)	2.088 ± 0.034	2.09 ± 0.081	1.931 ± 0.090	1.870 ± 0.065	1.953 ± 0.050	0.102

AST: aspartate Aminotransferase

ALT: alanine aminotransferase

**Table 5:** Hematological response

	Control	CV	CV + ZnO-NPs	Ch + Se-NPs	CV + ZnO-NPs + Se-NPs	*P* values
RBCs (10^6^ /mm^3^)	2.50 ± 0.198 ^**b**^	3.06 ± 0.365 ^**ab**^	2.66 ± 0.181 ^**ab**^	2.65 ± 0.227 ^**ab**^	3.75 ± 0.385 ^**a**^	0.046
HB (g/100mL)	8.588 ± 0.253 ^**b**^	9.900 ± 0.712 ^**ab**^	9.903 ± 0.462 ^**ab**^	9.617 ± 0.612 ^**ab**^	11.153 ± 0.675 ^**a**^	0.042
PCV (%)	23 ± 1.817	28 ± 3.225	25 ± 1.780	25.333 ± 2.082	33.25 ± 3.262	0.084
MCHC	37.98 ± 1.783	36.44 ± 2.082	39.88 ± 0.963	38.103 ± 0.823	34.185 ± 1.539	0.173
MCH	34.98 ± 1.656	33.48 ± 2.086	37.50 ± 0.907	36.54 ± 1.003	30.515 ± 1.768	0.067
MCV	92.105 ± 2.23	91.93 ± 2.838	94.047 ± 0.593	95.88 ± 1.262	98.15 ± 2.252	0.298
WBC*	42.103 ± 2.6	49.53 ± 3.76	39.897 ± 4.081	53.053 ± 6.426	50.428 ± 3.478	0.162
Basophils*	1.438 ± 0.33	1.648 ± 0.382	0.897 ± 0.387	1.33 ± 0.478	0.923 ± 0.339	0.539
Monocytes*	3.405 ± 0.46	3.995 ± 0.25	2.793 ± 0.150	3.58 ± 0.369	4.10 ± 0.513	0.191
Lymphocytes*	27.25 ± 1.62	31.868 ± 2.27	27.263 ± 3.386	36.5 ± 5.227	33.328 ± 1.420	0.158
Heterophils *	8.863 ± 0.43	10.403 ± 1.38	7.15 ± 0.161	10.093 ± 1.586	10.35 ± 1.497	0.318
H/L ratio^**^	0.328 ± 0.04	0.328 ± 0.03	0.275 ± 0.035	0.275 ± 0.038	0.306 ± 0.033	0.585

*(10^6^ / ml )

** Heterophils / Lymphocytes ratio

### Antioxidant and immune responses

Dietary supplementation of CV, CV + ZnO-NPs, CV + Se-NPs, and their combination distinctly modulated the antioxidant and immune responses (Table 6). Prominent alterations in the antioxidant levels were noticed. A marked increase in the serum GPX level was measured in all supplemented groups compared with the control one (*P* < 0.05). The highest GPX levels were found in the case of combining CV with both ZnO-NPs and Se-NPs (*P* < 005). For the SOD level, the highest level was reported in the CV dietary-supplemented group compared to the control and other supplemented groups (*P* < 0.05). Minor increases in the SOD level were found in the other supplemented groups (*P* > 0.05). Moreover, the levels of CAT displayed minor changes following the CV, ZnO-NPs, and Se-NPs dietary supplementations (*P* > 0.05). Only slight increases in the CAT levels were found in the supplemented groups compared to the control with the highest increases noticed in the CV and CV + ZnO-NPs + Se-NPs groups.

**Table 6:** Antioxidant and immune responses

	Control	CV	CV + ZnO-NPs	Ch + Se-NPs	CV + ZnO-NPs + Se-NPs	*P* values
GPX (mU/ml) ^(1)^	10.145 ± 0.081 ^**b**^	12.238 ± 1.136 ^**ab**^	11.667 ± 0.629 ^**ab**^	11.387 ± 0.338 ^**ab**^	12.58 ± 0.971 ^**a**^	0.01
SOD (U/ml )^(2)^	21.628 ± 1.079 ^**b**^	30.245 ± 1.434 ^**a**^	23.053 ± 1.533 ^**b**^	22.387 ± 1.178 ^**b**^	20.387 ± 0.772 ^**b**^	< 0.001
CAT (U/l) ^(3)^	10.145 ± 0.081	12.238 ± 1.136	11.667 ± 0.629	11.387 ± 0.338	12.58 ± 0.971	0.229
Phagocytic activity	10.908 ± 0.401	11.134 ± 1.109	11.467 ± 0.292	13.51 ± 1.320	11.543 ± 0.540	0.275
Phagocytic index	1.883 ± 0.063 ^**b**^	1.583 ± 0.128 ^**b**^	1.950 ± 0.100 ^**a**^	1.948 ± 0.068 ^**a**^	1.99 ± 0.026 ^**a**^	0.012
Lysozyme (µg/mL)	10.115 ± 0.190	11.023 ± 1.473	12.028 ± 0.597	10.588 ± 0.363	10.937 ± 0.246	0.152

Glutathione peroxidase enzyme, (2): superoxide dismutase enzyme, (3): Catalase enzyme

Phagocytic activities were not altered following the dietary supplementation (*P* > 0.05). Whereas the phagocytic index was significantly changed; significant increases were noticed in the case of combining CV with ZnO-NPs + Se-NPs or both compared to the control and CV only (*P* < 0.05). The serum LZM level was not modulated with the dietary supplementation of CV with ZnO-NPs, Se-NPs, or both (*P* > 0.05). Slight elevations in its level were measured in the case of CV and CV + ZnO-NPs compared to the other groups.

### Histological features of jejunum, liver, and spleen

**Fig. 1:**
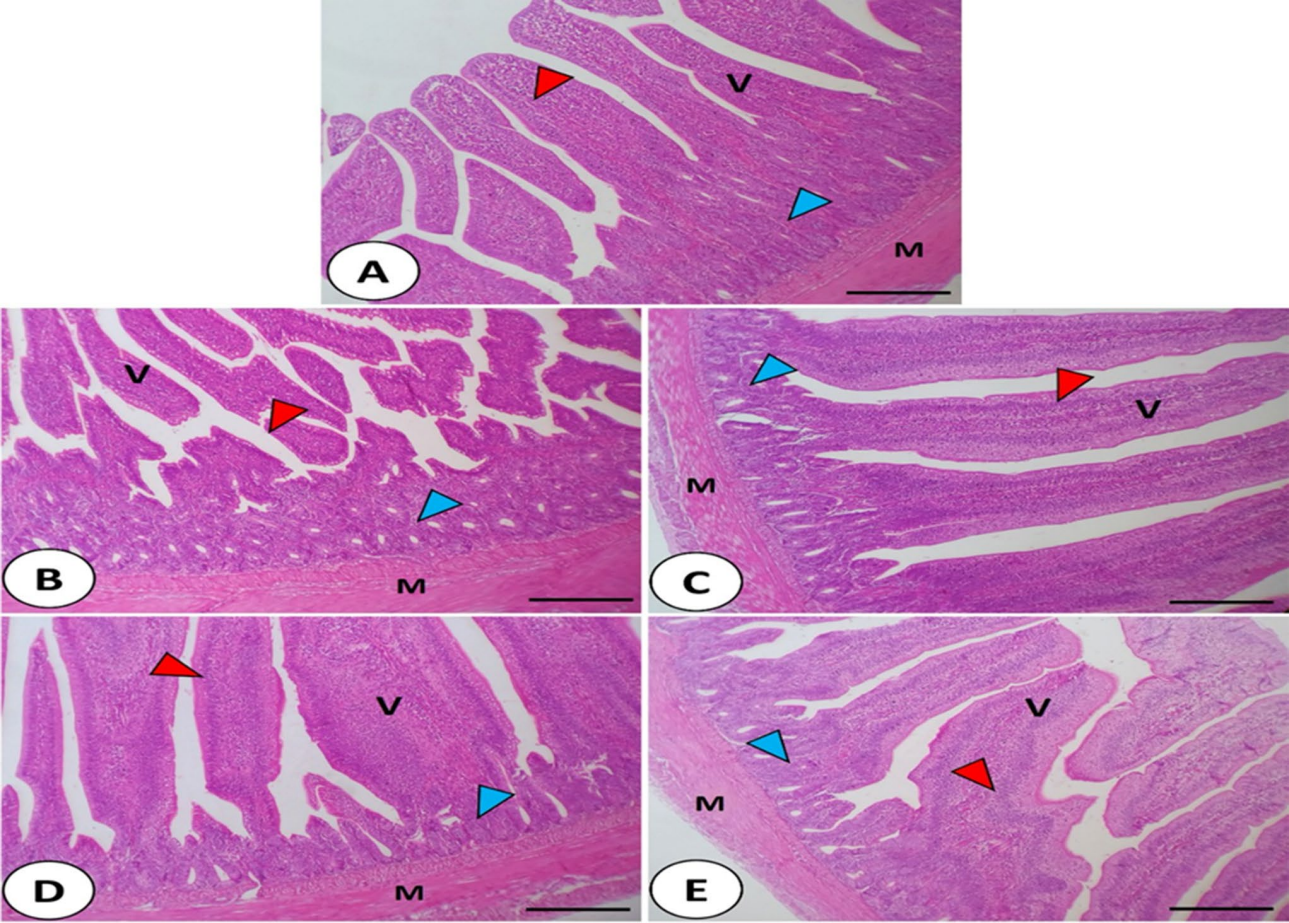
Histomicrograph of the chicken jejunum in the control group (**A**) as well as other treated groups; CV (**B**), CV + ZnO-NPs (**C**), CV + Se-NPs (**D**), and CV + ZnO-NPs + Se-NPs (**E**). The histological structure of the jejunum in all groups showed normal appearance of intestinal villi (V), propria submucosa with simple tubular glands (blue arrowhead) and tunica muscularis mucosae (M) which covered externally by serosa. However, the supplemented groups with chlorella and/or its combinations showed better morphological appearance of intestinal villi with well-arranged simple columnar epithelium (red arrowhead). Stain H&E. Bar 150 μm. **F**, **G**, and **H** donate the differences in villi length, villi width, and crept depth, respectively between the groups

**Fig. 2:**
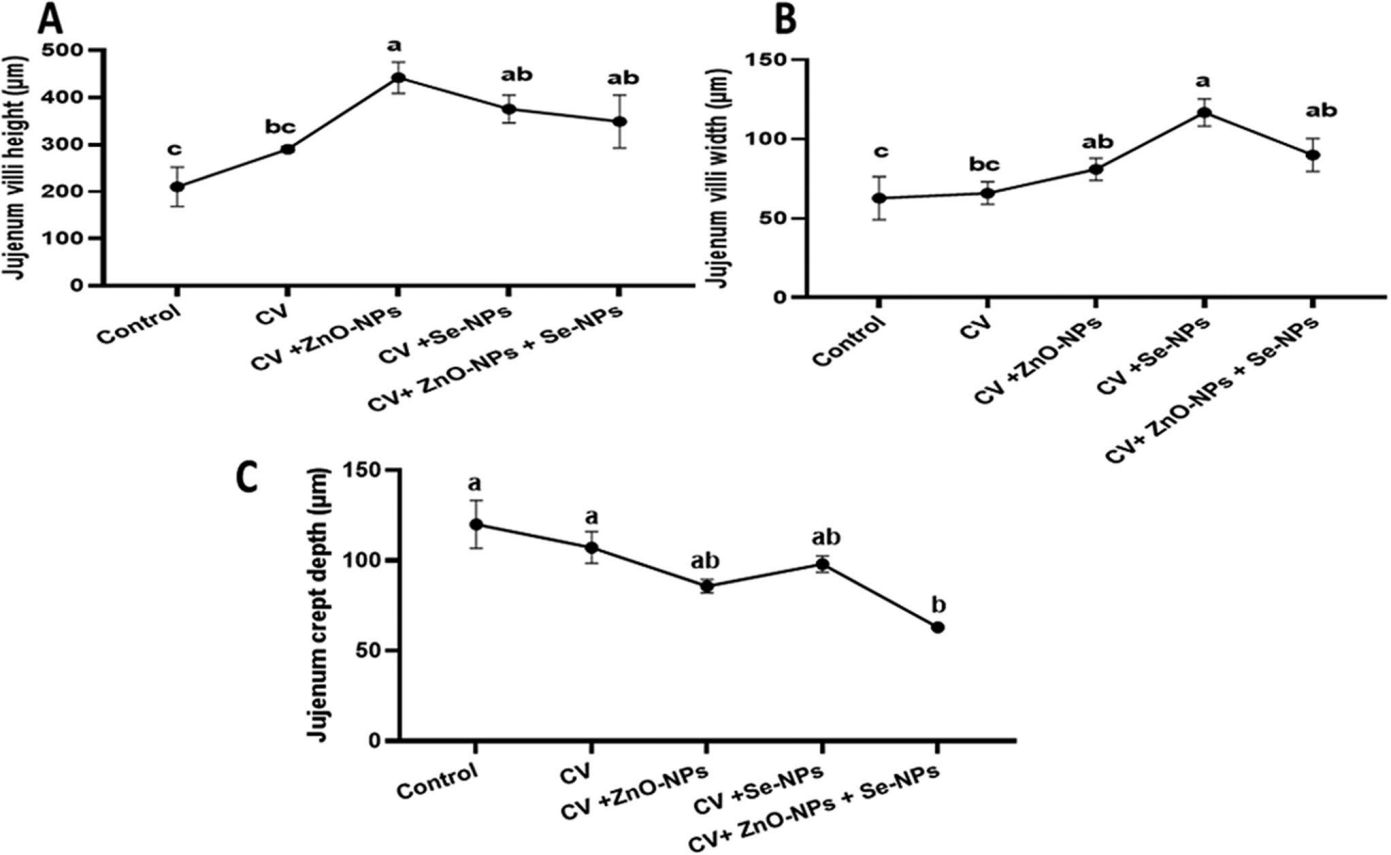
The quantitative morphometric analysis of the jejunum in response to CV, and/or ZnO-NPs, and Se-NPs dietary supplementation. **A** shows that intestinal villi length, **B** represents that intestinal villi width, while **C** demonstrates the crypt depth. Different lowercase letters indicate statistical significance at *P* < 0.05

**Fig. 3:**
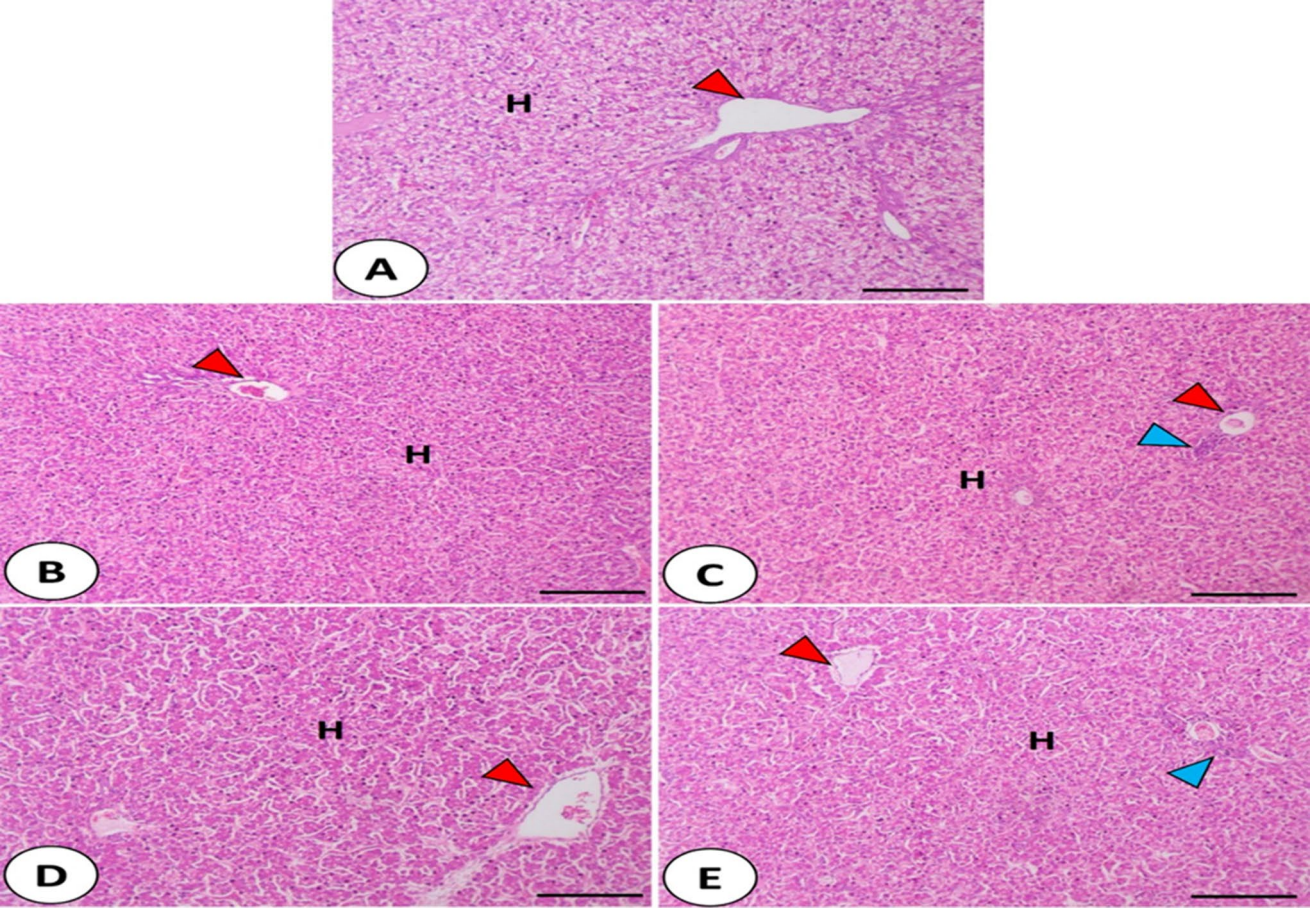
Histomicrograph of the chicken liver in the control group (**A**) as well as other treated groups; CV (**B**), CV + ZnO-NPs (**C**), CV + Se-NPs (**D**), and CV + ZnO-NPs + Se-NPs (**E**). The construction of the liver in the control chicken showed a normal appearance of hepatocytes (**H**) separated by blood sinusoids around the central vein (red arrowhead). All groups rather than control one revealed improved appearance of hepatic parenchyma with glycogen deposition in addition to perivascular immune cell infiltration (blue arrowhead). Stain H&E. Bar 100 μm

The impact of CV dietary supplementation with or without ZnO-NPs, Se-NPs, or both on the histological structure of the jejunum, liver, and spleen was examined (Figs. 1 and 2, and Fig. 3, respectively). In the control non-supplemented group (Fig. 1.A), normal histological features of jejunum were found. Normal appearance of intestinal villi with simple columnar absorptive cells, propria submucosa with simple tubular glands, and lamina muscularis mucosae was noticed. The tunica muscularis was formed of inner circular and outer longitudinal smooth muscle fibers which covered externally by serosa. However, the supplemented groups (Fig. 1. B-E) with CV, and its combinations with ZnO-NPs, Se-NPs, and both showed better morphological appearance of intestinal villi that presented by well-arranged enterocytes, and wide absorptive area of intestinal villi. The quantitative morphometric analysis of the examined intestinal segment revealed increases in the intestinal villi height, and width (Fig. 2.A-B, respectively) in the supplemented groups especially in the case of CV + ZnO-NPs (442.3 μm), CV + Se-NPs (375.6 μm), and CV + ZnO-NPs + Se-NPs (348.8 μm) compared with CV only (290.3 μm) and the control (210.2 μm) (*P* < 0.05). However, a significant reduction in the jejunal crept depth (Fig. 2.C) was reported when the CV was combined with ZnO-NPs + Se-NPs (63.1 μm) compared with the other supplemented and control (120.1 μm for control, 107.2 μm for CV only, 85.84 μm for CV + ZnO-NPs, and 98.08 μm for CV + Se-NPs) (*P* < 0.05).

The construction of the liver in the control chicken (Fig. 3.A) showed a normal appearance of hepatocytes separated by blood sinusoids around the central vein. All groups rather than control (Fig. 3.B-E) revealed an improved appearance of hepatic parenchyma with glycogen deposition in addition to perivascular immune cell infiltration.

**Fig. 4:**
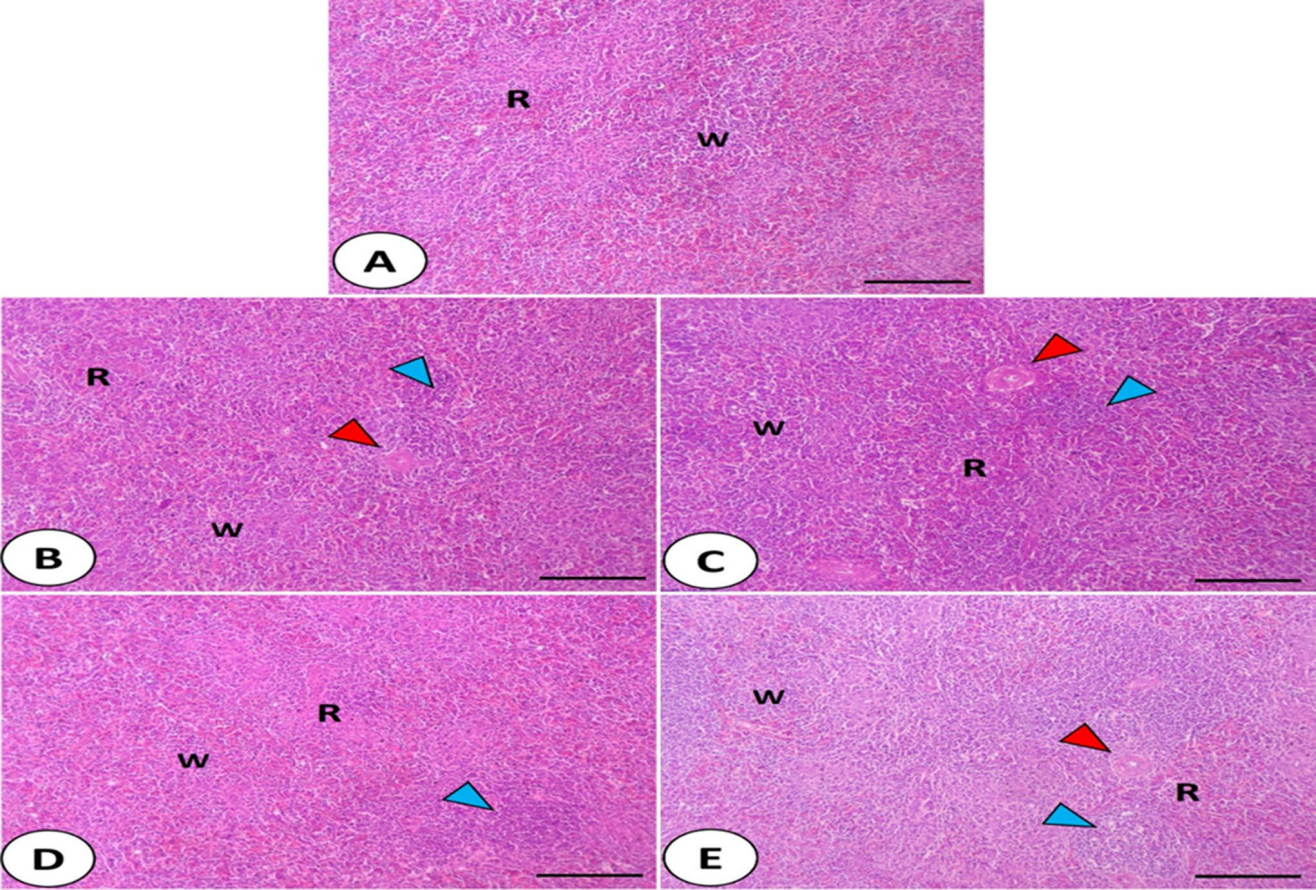
Photomicrograph showed the splenic architecture presented the normal appearance of red and white pulps in all experimental groups. In addition, there were lymphoid nodules near the sheathed arteries in the treated groups (**B**-**E**) as well as diffuse lymphocytic infiltration in the group supplemented with chlorella and Zn O-NPs + Se-NPs (**E**)

The splenic architecture (Fig. 4.A-E) presented the normal appearance of red and white pulps in all experimental groups. In addition, there were lymphoid nodules near the sheathed arteries in the treated groups as well as diffuse lymphocytic infiltration in the group supplemented with chlorella and Zn O-NPs + Se-NPs.

### Gene expression response

**Fig. 5:**
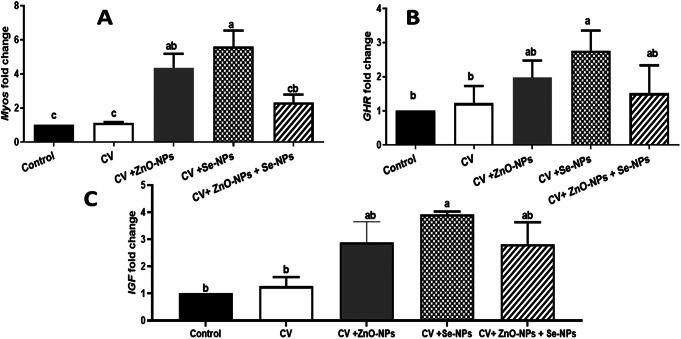
The relative expression levels of *myostatin (MYOS)*,* growth hormone receptor (GHR)*, and *insulin-like growth factor (IGF)* following in the control group (BD only) as well as other treated groups; CV, CV + ZnO-NPs, CV + Se-NPs, and CV + ZnO-NPs + Se-NPs. Different lowercase letters indicate statistical significances at P values 0.0009, 0.0199, and 0.0136 for MYOS, GHR, and IGF, respectively

The relative expression levels of growth-regulating genes in muscle such as *myostatin (MYOS)*,* growth hormone receptor (GHR)*, and *insulin-like growth factor (IGF)* were significantly modulated by the dietary supplementation of CV, and its concurrent combination with ZnO-NPs (CV + ZnO-NPs), Se-NPs (CV + Se-NPs) or both (CV + ZnO-NPs + Se-NPs). In this regard, CV dietary supplementation only failed to alter the *MYOS* (Fig. 5.A) mRNA level compared to the control (1.133 fold compared to 1 fold, respectively) which fed the BD only (*P* > 0.05). Whereas combining CV dietary supplementation with ZnO-NPs (4.347 folds), Se-NPs (5.602 folds) or both (2.324 folds) significantly upregulated the MYOS mRNA expression level (*P* < 0.05). The separate combination of CV with either ZnO-NPs or Se-NPs, especially the Se-NPs, induced noticeable higher mRNA levels (5.602 folds) (*P* < 0.05) compared to the others. For *GHR* and *IGF* genes (Fig. 5.B &C, respectively), the dietary supplementation of CV only or its combination with ZnO-NPs, Se-NPs, or both significantly increased their mRNA levels in muscle tissue (*P* < 0.05). Again, the separate combination of Se-NPs with CV induced the highest levels of *GHR* and *IGF* genes (2.759, and 3.917 folds, respectively) (*P* < 0.05).

**Fig. 6:**
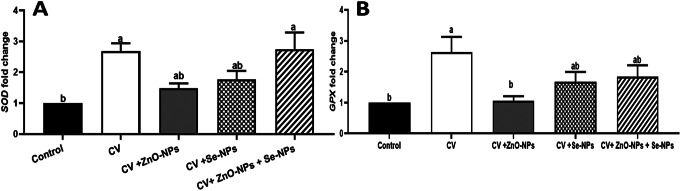
The mRNA copies of *SOD*, and *GPX* antioxidant genes (**A**,** B**, respectively) following in the control group (BD only) as well as other treated groups; CV, CV + ZnO-NPs, CV + Se-NPs, and CV + ZnO-NPs + Se-NPs. Different lowercase letters indicate statistical significances at P values = 0.0090 and 0.0274 for SOD and GPX, respectively

The mRNA copies of *SOD* and *GPX* antioxidant genes (Fig. 6. A, & B, respectively) were also, modulated by the CV, CV + ZnO-NPs, CV + Se-NPs, and CV + ZnO-NPs + Se-NPs dietary addition. For the estimated genes (*SOD*, and *GPX)*, the Cobb birds supplemented with CV ( 2.690 folds for SOD & 2.639 for GPX ), CV + ZnO-NPs (1.481 folds for SOD & 1.065 for GPX), CV + Se-NPs (1.760 folds for SOD & 1.667 for GPX), and CV + ZnO-NPs + Se-NPs (2.744 folds for SOD & 1.8.38 for GPX ) exhibited significantly increased mRNA copies compared with those fed the BD only (*P* < 0.05). The CV independently (CV) and with both ZnO-NPs and Se-NPs (CV + ZnO-NPs + Se-NPs) induced the highest expression levels for the *SOD* compared to the CV + ZnO-NPs or control (*P* < 0.05). For *GPX*, the separate supplementation of CV stimulated the largest number of mRNA copies compared with the other treatments and control (*P* < 0.05).

**Fig. 7:**
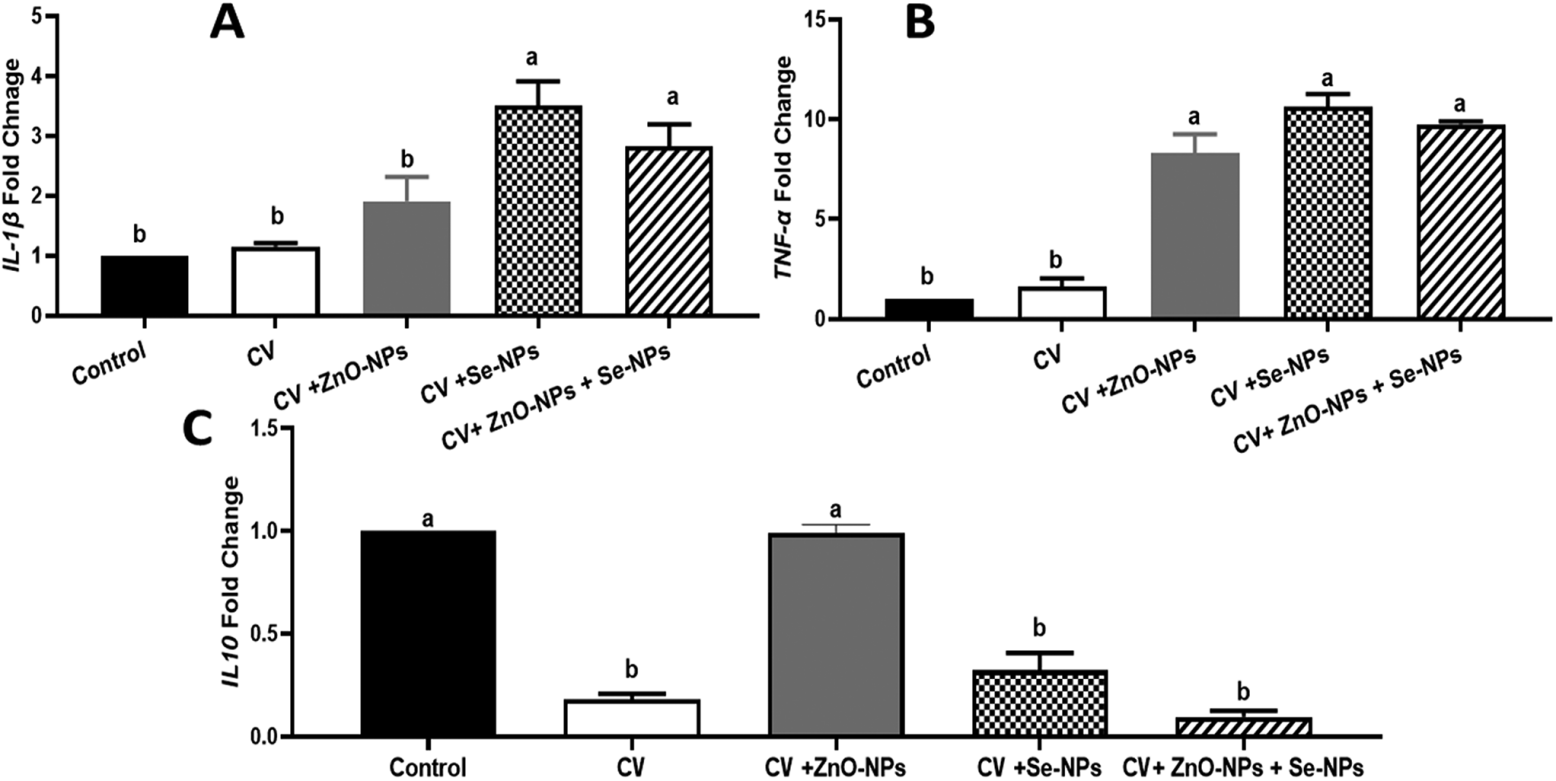
*IL-1B*,* TNF-ά* and *IL-10* mRNA levels following in the control group (BD only) as well as other treated groups; CV, CV + ZnO-NPs, CV + Se-NPs, and CV + ZnO-NPs + Se-NPs. Different lowercase letters indicate statistical significances at P values 0.0006, < 0.0001, and < 0.0001 for IL-1B, TNF-ά, and IL-10, respectively

The transcriptomic levels of the non-specific immune response-related genes such as *IL1-β*,* TNF-α*, and *IL10* (Fig. 7. A, B, & C, respectively**)** were also, assessed. For *IL1-β*, merging the CV with Se-NPs and Se-NPs plus Zn O-NPs significantly increased its mRNA copies (3.508 & 2.931 folds, respectively) compared with BD, CV only, and CV + Zn O-NPs (1.00, 1.152, and 1.911 folds, respectively) (*P* < 0.05). Similarly, all the combining supplementation of CV with Zn O-NPs, Se-NPs, and both significantly upregulated the *TNF-α* mRNA levels (8.299, 10.65, and 9.760 folds, respectively) (compared with the CV (1.618 fold or BD (1.00 fold) (*P* < 0.05). However, for *IL-10* all the used dietary supplements except CV + Zn O-NPs induced marked down regulation of its mRNA levels (0.178, 0.9881, 0.320, and 0.094-fold, respectively) (*P* < 0.05).

## Discussion

In this study, obvious improvement of growth performance confirmed by increasing the final body weight, and body gain as well as improving the FCR were recorded in the case of the concurrent supplementation of CV with ZnO-NPs, or Se-NPs. These improving effects were higher in the separate combination of CV with ZnO-NPs or Se-NPs compared with the concurrent supplementation which possibly confirms the synergistic effects of CV with the separate supplementation of zinc or selenium nanoparticles. However, increasing the sample size and including more birds for recording the growth parameters are highly recommended in future investigations.

Zinc and selenium are crucial microminerals for the growth, antioxidant, and immune performance of birds plus they are crucial co-factors for many enzymes involved in carbohydrate metabolism (Alagawany et al. 2021). Besides, the emergence of nanotechnology and the availability of their salts in nanoforms; ZnO-NPs and Se-NPs increase their bioavailability and absorption which guarantee the release of sufficient levels compared with their supplementation in the organic and inorganic salts (El-Kassas et al. 2019). Therefore, the improved growth performance as a result of CV combination with ZnO-NPs or Se-NPs may be correlated with the higher bioavailability of Zn and Se particles and increasing their concentration (Ševčíková et al. 2006). Both Zn and Se are crucial in regulating the levels of other micro minerals such as copper which is a co-factor in several metalloenzymes regulating hemoglobin formation, and carbohydrate metabolism (El-Kassas et al. 2019; Olechnowicz et al. 2018). The latter effect possibly helps in the digestion of CV via degrading its insoluble carbohydrates-rich cell wall and consequently, releasing its high nutrient content. However, the absence of digestibility and bioavailability measurements is one of the critical limitations of this study. Therefore, future investigations to assess the CV digestibility and bioavailability following its separate and concurrent combination with ZnO-NPs or Se-NPs are recommended. Furthermore, CV is rich in essential amino acids (60%) and other bioactive compounds such as PUFAs (10%), polysaccharides (20%), volatile and phenolic constituents, vitamins, and sterols (Abdelnour et al. 2019). Besides, CV does not produce any toxic metabolites or any anti-nutritional factors (Abdelnour et al. 2019). All these properties of CV might regulate and improve a bird’s growth performance. Similar outcomes were described by (Alfaia et al. 2021) who stated an improvement in the broiler’s growth performance following the dietary combination of carbohydrate-active enzymes with *Chlorella vulgaris*. Moreover, the fermentation helped the CV to improve the commercial Pekin duck’s and laying hen’s growth performance via enhancing the flavour of food and increasing feed intake but not efficient nutrient utilization (Oh et al. 2015; Zheng et al. 2012). Our findings support these explanations, in this regard, higher feed consumptions were measured in the case of CV combination with ZnO-NPs or Se-NPs despite the negative effects of CV on food palatability because of the distinct algal odour of CV (Abdelnour et al. 2019). This may indicate that the dietary mixture of CV-ZnO-NPs or CV-Se-NPs improved food palatability and increased feed intake. Therefore, further studies are recommended to investigate how the CV dietary combination with ZnO-NPs or Se-NPs regulates birds’ feed intake, especially with the less development of taste sensors in poultry compared to mammals (Oh et al. 2015). In contrast, the study of (Cabrol et al. 2022) reported that the partial dietary replacement of soybean meal with CV by 15% and 20% lowered broiler’s growth. Also, its supplementation at 0.5% decreased FI and body gain due to lowering the food palatability with the higher levels of the dietary incorporation of CV (Abdelnour et al. 2019; Kotrbáček et al. 2015). These contradictory findings could be attributed to the inclusion and supplementation levels, differences in experimental designs, and the form of CV (Roques et al. 2022).

The increased growth performance following the combined supplementation of CV with ZnO-NPs and/or Se-NPs might be also, explained by the improved histological features of the liver, and intestine. In this regard, the highest villi lengths were measured for the broiler supplemented with the CV with either ZnO-NPs or Se-NPs. Increasing intestinal villi length increases the absorption of nutrient precursors such as those of protein, glucose, and lipids which in turn enhance bird’s growth (Kang et al. 2017; Kirrella et al. 2023a, b). Besides, the improved growth performance as a result of improving the intestinal architecture might be associated with the antibiotic effects of CV and improving the intestinal microflora due to its high contents of chlorellin (Oh et al. 2015). Similarly, the results of (Kang et al. 2017; Mirzaie et al. 2020) displayed significant increases in the intestinal morphometric indices such as the height of intestinal villi and/or crypt depth of broilers fed CV by-products. However, (Roques et al. 2022) documented no changes in the broiler’s jejunum villus height and/or crypt as a result of CV biomass feeding. These discrepant results might be associated with the differences in the studied intestinal section and the age of the bird at investigation (Roques et al. 2022). Besides, the improved growth in the case of combined supplementation of CV and ZnO-NPs or Se-NPs might be linked with the increased glycogen deposition in the liver tissue. High glycogen increases glucose levels through glycogen catabolism which possibly stimulates spare body protein (Zhai et al. 2011).

Moreover, the obtained results of the growth-regulating genes, *MYOS*,* GHR*, and *IGF* in the case of combining the CV with ZnO-NPs and/or Se-NPs might support the improved bird’s growth. The highest mRNA levels of these genes were reported for the birds that received a diet supplemented with CV and/or ZnO-NPs & Se-NPs compared to CV only and control diet. Increasing the mRNA levels of these genes could confirm the improved growth performance because of the combined supplementation of CV with ZnO-NPs and/or Se-NPs. Similar results reported increases in broilers’ growth because of the upregulating of transcriptomic levels of *GHR*,* IG*, and *MYOS* genes (El-Naggar et al. 2019; Roelfsema et al. 2018; Sakr et al. 2020).

Besides, the modulations of the growth performance of broilers following the CV + ZnO-NPs or CV + Se-NPs nanocomposites, the biochemical profile was also, altered with significant increases in Hb, and RBCs. The increases in the broiler’s hematological parameters are possibly due to the content of the CV, like other microalgae, of phycocyanin and polysaccharide which increase the bioavailability of iron through reducing the ferric ions into ferrous (Abdel-khalek et al. 2023). Additionally, the reported highest levels in the case of CV + ZnO-NPs and CV + Se-NPs may be correlated with the presence of Zn and Se which are cofactors of many enzymes that regulate the enzymatic and physiological processes involved in the nutrients’ metabolism and digestion in the digestive tract (Hidayat et al. 2020). Similar results supported our findings such as the study of (Abdel-khalek et al. 2023) which reported improvements in RBCs, Hb, and WBC profiling following CV feeding in goats.

The antioxidant and immune responses of broilers were also, modulated because of the separate and concurrent dietary supplementation of CV, ZnO-NPs, and/or Se-NPs. Noticeable increases in the antioxidants enzymes activities such as the GPX, SOD, and CAT were found in the case of CV + Se-NPs and CV + ZnO-NPs + Se-NPs compared with the control diet. This antioxidant response following the dietary supplementation of CV, ZnO-NPs, and/or Se-NPs might be correlated with the functional bioactive ingredients of CV. CV like other microalgae is rich in active and functional ingredients such as β-carotene, phenolic acid, γ-linolenic acid, flavonoid, phycobi-liproteins (β-phycocyanin and C-phycocyanin), and chlorophylls which improve bird’s redox system and health through scavenging the excessive production of reactive oxygen species (ROS), preventing lipid peroxidation, and improving the production of key antioxidant enzymes such as GPX, SOD and CAT (Abdelnour et al. 2019). Besides, the concurrent supplementation of ZnO-NPs and /or Se-NPs improved the reported antioxidant response indicating the synergistic effect between the CV and ZnO-NPs and /or Se-NPs where CV is a rich source of zinc and selenium. The Zn and Se are essential trace elements that improve the antioxidant synthesis (Olechnowicz et al. 2018; Ševčíková et al. 2006). These findings could be confirmed by the changes in the gene expression levels of antioxidant genes such as *SOD* and *GPX* which displayed marked up-regulations following CV and/or ZnO-NPs and Se-NPs dietary supplementation. These reported antioxidant responses of the separate and concurrent dietary supplementation of CV, ZnO-NPs, and/or Se-NPs agreed with the findings of (El-Bahr et al. 2020; Park et al. 2018) which stated increases in the serum SOD and GPX levels following the dietary supplementation of *Spirulina platensis*, *Chlorella vulgaris*, and *Amphora coffeaformis* to broilers, respectively. Additionally, (Subhani et al. 2018) reported dose-dependent increases in the antioxidant enzyme activities of SOD, CAT, and GSH-Px following the dietary supplementation of *Chlorella pyrenoidosa* to aflatoxin B1 exposed broilers. However, there is a lack of information on the antioxidant effects of the separate and combined dietary supplementation of CV, ZnO-NPs, and/or Se-NPs in poultry, where most of the previous studies proved the antioxidant characteristics of CV only in birds. Therefore, this feeding trial could confirm, for the first time, that the dietary combination of CV, ZnO-NPs, and/or Se-NPs possibly improves the antioxidant status of birds by increasing the antioxidant enzyme activities. This effect is perhaps because of the role of Zn and Se in releasing the CV-bioactive ingredients besides, the effectiveness of both Zn and Se as crucial trace minerals in increasing the antioxidant enzyme activities. Nevertheless, the absence of stress exposure in this study is one of its limitations hence including a stress factor in this experiment would support the study outcomes. Thus, future investigations are recommended to explore how the synergistic combination of CV with ZnO-NPs, and/or Se-NPs modulate the antioxidant and immune response under stress conditions.

The non-specific immune response of broilers was also, altered in response to the dietary supplementation of CV, ZnO-NPs, and/or Se-NPs. For instance, increases in phagocytic and lysozyme activities were reported. These conclusions might prove the efficient role of CV in promoting the bird’s immunity, health, and welfare (Abdelnour et al. 2019). The previous reports (An et al. 2016; Kang et al. 2017) presented increases in the humeral immune response manifested by increasing the plasma IgG and IgM contents in response to CV supplementation due to the high levels of the antioxidants and omega-3 PUFAs in CV. The previous studies claimed the improvement of CV in rodents’ and chicks’ immune response to increasing the cytokines production such as IL-2 and IL-4 plus enhancing the phagocytic activities, respectively (Kotrbáček et al. 2015; Kotrbáček et al. 1994). These responses may be attributed to the contents of CV, like other microalgae, of polysaccharides such as β-glucan, β carotene, and vitamin B1 that have pivotal immunoregulatory roles in the inflammatory and immune responses of animals, birds, and even humans (Abdelnour et al. 2019). Furthermore, combining the ZnO-NPs and/or Se-NPs with CV might assist the effects of CV hence; Zn and Se are crucial trace elements in the modulation of immune response (Olechnowicz et al. 2018). Moreover, the increases of phagocytic and lysozyme activities in response to the ZnO-NPs and/or Se-NPs combination with CV are possible because of increasing the production of γ-interferon that promotes the activity of macrophages and immune cells (Abdelnour et al. 2019).

## Conclusion

Collectively, the dietary supplementation of CV with ZnO-NPs and /or Se-NPs at 1 g/kg diet resulted in significant enhancements of the broiler’s body weight, gain, and FCR. The improved growth performance was linked with the up-regulation of mRNA levels of some growth-regulating genes such as *MYOS*,* GHR*, and *IGF* with marked enhancements of intestinal morphometric indices and distinct modulations of blood biochemical. Also, obvious increases in the antioxidant enzyme activities of SOD, GPX, and CAT were accompanied by up-regulation of their transcriptomic levels were reported. Moreover, there was an alteration in the broiler’s non-specific immunity characterized by improving the phagocytic and lysozyme activities. All the obtained results were prominent in the case of the concurrent supplementation of CV with ZnO-NPs and /or Se-NPs confirming the synergistic mechanisms of CV with ZnO-NPs and /or Se-NPs. Therefore, the dietary combination of CV with ZnO-NPs and /or Se-NPs could be recommended in broiler diets to enhance their growth, antioxidant, and immune response. However, further future investigations to examine the effect of the dietary combination of CV with ZnO-NPs and /or Se-NPs on the digestibility and bioavailability of CV and including more birds and a stress factor in the study design are recommended.

## Data Availability

The authors acknowledge that the data presented in this study will be available at a reasonable request.
